# Obstetrics and Gynecology

**Published:** 1893-04

**Authors:** 


### OBSTETRICS AND GYNECOLOGY.

EDITED BY DR. VIRGIL O. HARDON.

### EXTRA-UTERINE PREGNANCY.

A. Martin, in the consideration of extra-uterine pregnancy before the First International Congress for Gynecology, held in Brussels, September 15, 1892 (*Wiener Medizinische Wochenschrift*, No. 41, 1892), drew the following conclusions:

(1) The etiology of extra-uterine pregnancy is yet veiled in the deepest darkness. The question cannot be solved before the physiology of pregnancy is explained.

(2) Most frequently the egg is implanted in the tube. Ovarian pregnancies are less seldom, and the abdominal insertion of the egg is doubtful.

(3) The diagnosis of ectopic pregnancy is a diagnosis of probability, with the exception of those cases where we observe the development of the fetal membranes outside the cavity of the uterus, or can determine the development of an intrauterine decidua without a separable chorion, or when we discover the fetus.

(4) The development of an extrauterine pregnancy ends seldom in retrogade metamorphosis (Lithopædian, mummification) without the occurrence of some event.

(5) Usually the death of the ovum takes place through hemorrhage in the fetal membranes or in the ovum itself. The blood finds entrance to the abdominal cavity through the ostium of the tube (tubal abortion), or through rupture of the tube in its continuity; it is found either in the peritoneal cavity or in the ligamentum latum. The hemorrhage seldom ceases. Death usually occurs from anemia or peritonitis, the true nature of which is unexplained. The ectoptic pregnancy should be regarded as a dangerous neoplasm, and handled accordingly. The cases which progress to the end of pregnancy are so very rare that regard for the child is entirely secondary to that of the mother.

(6) The conclusion is that the operative treatment shall, so soon as possible in all forms of ectopic gestation, be instituted. The fetal sac must be shelled out when possible. The treatment by subcutaneous injections of morphia produces very slow healing. The electric treatment cannot yet be judged according to its true worth, as the cases as yet treated by this method have not been sure in their diagnosis.—*Univ. Med. Mag.*

---

### GONORRHŒAL INFECTION IN WOMEN AND ITS CONSEQUENCES.

Bumm (*Muench. Med. Woch.*, 1891, Nos. 50 and 51) writes at length on this subject. His experiences show that gonorrhœal infection does not pass readily to the tubes and peritoneum. The external and internal os form usually a barrier against the entrance of the gonococci into the uterine cavity. This barrier may be broken down by the process of menstruation, by the puerperium and by therapeutic manipulations. The infection of the uterine cavity manifests itself by high fever, chills, severe uterine colic, a sero-sanguinous flow, and later on by a purulent discharge. During the acute and chronic stages there is an extreme tenderness of the organ which is frequently excited anew by fresh exacerbations of the inflammatory process. If the process attacks the tubes it is not long before the peritoneum is also involved. The inflammatory process in these structures does not present a true gonorrhœal form, for the gonococci thrive but poorly on serous surfaces ; it partakes rather of a septic form as the author has shown experimentally by inoculations into the peritoneum of rabbits. He does not believe that the infection passes to the endometrium and tubes in more than a third of the cases ; in the remaining two-thirds it is limited to the cervix and urethra. It is difficult to collect reliable statistics on this subject, for in venereal clinics only fresh cases are seen, which are soon lost sight of, and in gynecological clinics there is an accumulation of the serious cases, and lighter forms of the disease are likely to be overlooked. A true picture can only be obtained when cases of all degrees of infection are observed until they are cured, or for a long time. The author analyzed fifty-five cases in his experience which embody the latter conditions. Of these fifty (91 per cent.) had gonorrhœal urethritis ; forty-one (74 per cent.) had gonorrhœal cervicitis, and only in eight (14 per cent.) was there an infection of the uterine cavity, and only in two (3.6 per cent.) were the tubes affected.

---

### UTERINE HEMORRHAGE AFTER MENOPAUSE.

Monod (*Nouv. Arch. d' Obstét. et de Gynéc.*) has observed several cases of obstinate hemorrhage in otherwise healthy women long after the menopause. Metritis is not rare in age. An interstitial fibroid, small enough to escape detection by the sound, yet near enough to the endometrium to keep up permanent congestion, may explain the bleeding. Mucous polypi may be hidden in the uterine cavity. The most important and most frequent cause of hemorrhage, as all admit, is cancer, which, in these cases, takes the form of epithelioma of the mucous membrane. Gusserow has collected 122 cases. This form of cancer is more especially limited to elderly patients, in contrast with cancer of the cervix. An "essential" metrorrhagia, with no local objective or subjective symptoms, has been observed. It may follow surgical injuries or other traumatisms. Mosse observed the appearance of a "false period," with all the usual phenomena, after tapping of an ovarian cyst, in a woman aged 67, who had ceased to menstruate for fifteen years. As the vessels of the senile uterus become atheromatous and the muscular tissue degenerates, it is not rare to see metrorrhagia in old women with thoracic, renal or hepatic disease. Obesity is not rarely associated with metrorrhagia in age, which disappears after "Banting" treatment. Moussous noted that in interstitial nephritis true uterine epistaxis may occur. Audebert, in the discussion on Monod's paper, pointed out that delayed menopause was not rare. He knew a lady, aged 88, who had never ceased to menstruate with perfect regularity. She remained in perfect health.—*Brit. Med. Jour.*

### VULVO-VAGINITIS IN CHILDREN.

*Conclusions.*—1. Vulvo-vaginitis is quite frequent in children, occurring in about one per cent. of all dispensary cases.

2. Most cases are infectious and in all probability of gonorrhœal origin.

3. Its gonorhœal nature has not yet been absolutely proven by bacteriological research.

4. The most frequent mode of infection is indirectly from the mother, or some other member of the family, by means of the general use of the same toilet articles, or by the children playing with each other's genitals. Occasionally the infection occurs from a case of ophthalmia, and in rare cases from infection at birth or from criminal action.

5. The affection runs a very prolonged course and usually does not produce much constitutional disturbance.

6. In rare cases it may lead to serious internal troubles, as salpingitis and pelvic peritonitis.

7. The diagnosis between the infectious and non-infectious varieties is only possible by means of the microscope; as a matter of precaution all cases should be treated as infectious.

8. The most efficient treatment consists in extreme cleanliness on the part of the child and its attendants, and local applications of a solution of nitrate of silver.

9. In children's hospitals, such cases should be isolated.—*Dr. T. W. Williams, Maryland Med. Journal.*

---

### SUBCUTANEOUS CHLOARL INJECTIONS IN PUERPERAL ECLAMPSIA.

Deshages, of New Orleans (*Abeille Medicin,* February 8, 1892), considers the subcutaneous injection of chloral a valuable method of its administration in puerperal eclampsia, where it is impossible to administer it by the mouth and there is a rectal intolerance. From his experience in several cases Deshages believes it to be the safest working method of administering chloral. In all cases an initial dose of from 7½ to 15 grains sufficed at once to quiet the patient and to lengthen the period between the seizures. The total quantity used was usually from 45 grains to 1 dram, given either in three doses at intervals of eight to ten hours, or following a large initial dose, hourly, 1½ to 3 grains. The largest quantity used was 75 grains. The effect of the large initial dose differs in individuals, according to the depth of the uremic intoxication, the condition of the os, the pains, and to the nearness of delivery. The injections are only to be used in comatose conditions, on account of the severe pain accompanying them, unless morphia or cocaine is added. The strength of the solution should not be greater than 1-10, and the syringeful should be very slowly injected.

Deshages has also used this method in the eclampsia of non-puerperal origin, with good result.

---

### UMBILICAL HERNIA IN THE FEMALE.

Dr. A. Dudley (*American Journal of Obstetrics*) says:

In the female the treatment of umbilical or ventral hernia by mechanical support rather than by radical operation is unwise

The radical operation, if properly done, is not more dangerous than laparotomy for any other purpose.

The use of a buried non-absorbent suture is attended with more or less risk.

To secure a good result it is necessary to have perfect apposition of the cut edges of the linea alba.

In using any form of suture which includes skin, cellular tissue and peritoneum with the linea alba, we cannot be sure of proper apposition of the latter.

The silver-wire suture, if adjusted as I have described, can be worn from three to five weeks without causing irritation.

The small sinuses leading down to the linea alba from the use of the silver canulæ over the wire sutures are an advantage rather than otherwise in fleshy women.

If operators who have this accident follow in the wake of their work would only report their cases, the profession at large would profit by it and greater efforts be made to prevent the occurrence.

### THE CÆSAREAN SECTION AND ITS SUBSTITUTE.

Dr. C. P. Noble (*American Journal of Obstetrics*) says:

Cæsarean section in typical cases is a safe operation.

It should be performed preferably before labor, and not later than the first stage of labor.

The classical operation is to be preferred to puerperal hysterectomy in typical cases, because it is equally if not more safe, and because it preserves the fertility of the woman.

Puerperal hysterectomy is to be preferred in certain atypical cases: (*a*) Cases, seen late, in which infection of the birth canal and atony of the uterus are to be feared. (*b*) Infected cases. (*c*) Cases complicated by large fibroid tumors of the uterus.

Symphyseotomy will probably supersede Cæsarean section done for the relative indication.

Embryotomy is no longer justifiable on the living, viable child as an elective operation.

---

### VOMITING OF PREGNANCY.

Dr. Weil recommends that ten drops of a twenty per cent. solution of menthol in olive-oil, dropped on powdered sugar and sugar sprinkled over it, be taken whenever nausea appears. By this means relief can be obtained, he says, in the most severe cases of apparently uncontrollable vomiting.

Dr. Neichtoube treats vomiting of pregnancy as follows:

℞. Cocaine sulphat., gr. xv.
Aq. dest., ℥ ii.

Take ten drops. If necessary repeat in an hour. If no effect is produced, repeat at the end of three hours. The following day give five or six drops three times, and keep it up until the vomiting ceases. At the same time apply to the vagina tampons covered with a two per cent. cocaine ointment.—*Med. Record.*

